# Analyzing the dynamic patterns of COVID-19 through nonstandard finite difference scheme

**DOI:** 10.1038/s41598-024-57356-9

**Published:** 2024-04-11

**Authors:** Abeer Aljohani, Ali Shokri, Herbert Mukalazi

**Affiliations:** 1 Department of Computer Science, Applied College, Taibah University, Medina, 42353 Kingdom of Saudi Arabia; 2 Department of Mathematics, Faculty of Science, University of Maragheh, Maragheh, 83111-55181 Iran; 3 Department of Mathematics and Statistics, Kyambogo University, Kampala, Uganda

**Keywords:** Epidemic, Unconditionally stable, Discretized, Differential equations, Establish, Overall behavior, Nonstandard finite difference scheme, COVID-19, Asymptomatic, Symptomatic, Computational biology and bioinformatics, Mathematics and computing

## Abstract

This paper presents a novel approach to analyzing the dynamics of COVID-19 using nonstandard finite difference (NSFD) schemes. Our model incorporates both asymptomatic and symptomatic infected individuals, allowing for a more comprehensive understanding of the epidemic's spread. We introduce an unconditionally stable NSFD system that eliminates the need for traditional Runge–Kutta methods, ensuring dynamical consistency and numerical accuracy. Through rigorous numerical analysis, we evaluate the performance of different NSFD strategies and validate our analytical findings. Our work demonstrates the benefits of using NSFD schemes for modeling infectious diseases, offering advantages in terms of stability and efficiency. We further illustrate the dynamic behavior of COVID-19 under various conditions using numerical simulations. The results from these simulations demonstrate the effectiveness of the proposed approach in capturing the epidemic's complex dynamics.

## Introduction

The COVID-19 pandemic has had a significant impact on both the global economy and public health systems. SARS-CoV-2, the virus responsible for COVID-19, was first identified in Wuhan, China, in late 2019. The virus rapidly spread worldwide, igniting a global pandemic with widespread infections, illnesses, and fatalities. Underdeveloped nations faced a disproportionate burden due to a confluence of factors, including limited healthcare resources, inadequate infrastructure, and stark socioeconomic inequalities. Developed nations, which have stronger healthcare systems have been better able to control and contain the spread^1,2^. It's important to remember that COVID-19 not only impacts the economy but also poses a threat to human life. Being in close proximity to an infected person increases the risk of contracting the virus, which spreads through saliva or mucus droplets. To protect citizens, many governments have implemented lockdown measures, while healthcare workers have committed to treating those affected by the disease^3,4^.

Certainly, it is important to acknowledge the commitment and effort put out by researchers, medical experts, and organizations in the battle against the COVID-19 pandemic. In order to stop the virus's spread and lessen its negative effects on the health of the world, scientists have worked hard to study the virus, create vaccinations, and put preventative measures in place. Effective pandemic management requires heeding advice from reputable organizations like the World Health Organization (WHO)^5,6^. In social situations where individuals share mouthpieces and hoses, smoking implements like water pipes, also known as hookahs or shisha, are frequently utilized. Due to the possibility of virus particles being transmitted through these common surfaces, this practice can in fact increase the spread of respiratory diseases, including Covid-19. When numerous people may be utilizing the same equipment in common settings, this manner of transmission is very worrisome^7–10^.

It is crucial to stay well-informed about the Coronavirus outbreak and take proactive measures to protect yourself and others. Recognizable symptoms of this virus include fever, cough, fatigue, vomiting, headache, diarrhea, difficulty breathing, and low white blood cell count. The incubation period of the virus can last up to 14 days. To safeguard yourself and others, it is essential to maintain a safe distance from infected individuals and seek medical attention if you experience any of its symptoms. You can obtain reliable information on the outbreak from reputable sources such as the WHO and CDC. Stay vigilant, stay safe, and prioritize your health and the well-being of others in your community^11,12^.

This study explores the steady-state flow of micropolar nanofluids subjected to the Hall current effect within parallel plates. We formulated an ODE system representing the governing flow dynamics using established formulae. Subsequently, explicit Runge–Kutta and Adams-based numerical solvers^13–17^ were employed to analyze the system's behavior. The differential equations obtained are solved numerically using the bvp4c method, which is a reliable technique. The fields of velocity, temperature, and concentration are found to be affected by various factors such as ferromagnetic interaction parameters, viscous dissipation, curie temperature, Weissenberg number, and thermal radiation. Additionally, the study examines and visually represents the thermal and velocity gradients^18–23^. Several scientists have focused on developing an effective strategy to control the transmission of the Coronavirus. Aims to control the spread of the virus by using some restrictions. Oke et al.^24^ proposed a mathematical model to analyze the Coronavirus outbreak in Africa. In their study, Yang et al.^25^ utilized a numerical model to investigate how vaccination affects the transmission of the Coronavirus in Africa. Their findings suggest that there is a decrease in the prevalence of the virus and a reduction in the significance of the smart crown infection^26–31^. Additionally, the study indicates an expansion in the event of recognition. Due to resistive connection, this model is totally different from the previous model which is discussed in this article^32–35^. Peter and associates^36^ used actual data analysis to assess the effects of different strategies for management on the spread of coronavirus among people in their investigation. The Coronavirus has been thoroughly investigated by other researchers^26,37–39^ utilizing mathematical models from many points of view, concentrating on local and global aspects, mathematical techniques, and stability theory.

Temesgen Duressa Keno and Hana Tariku Etana^40^ discussed how scientists analyze COVID-19 models using real data and assess the impact of different management strategies on virus spread. In this paper, we have studied the SEIHR model of COVID-19 and mathematically proved that the disease spreads less in the population. Our goal is to use an advanced NSFD scheme to validate various aspects of the continuous model, showcasing its sustainability and biological vitality. Our results demonstrate that this scheme is not only suitable for the model but also provides highly efficient and accurate outcomes. We have examined both local and global stability of disease-free equilibrium points and disease endemic equilibrium for the continuous model. Overall, developing the discrete NSFD model was driven by the desire to explore the qualitative behavior of the model and preserve its dynamic properties during numerical simulations. This approach is well-suited for analyzing global aspects of biological sustainability and other systemic approaches.

The paper is presented as follows: The mathematical model for the COVID-19 pandemic disease is described in “Various basic mechanisms characteristically type of a mathematical model” section. In “Equilibria and basic reproduction number ” section, gives information about the model stability and the basic reproduction number. The discrete NSFD scheme is created in “The NSFD Scheme” section, and in “The NSFD scheme's positivity and boundedness” section explores some fundamental features, including positivity and boundedness. Our findings demonstrate that the NSFD scheme is a reliable and effective technique that accurately represents the continuous model. The global stability of both equilibria is discussed in “Global stability of equilibria” section, while the local stability of each equilibrium is assessed using the Schur-Cohn criterion in “Local stability of equilibria” section. The numerical analysis and simulations we perform provide a strong evidence of theoretical findings that demonstrate the effectiveness of our results. All numerical results with graphical images are also discussed.

## Various basic mechanisms characteristically type of a mathematical model

This study utilizes the COVID-19 dynamical framework described in reference^39^, encompassing a system of five differential equations. Five compartments are utilized to categorize the complete population $M\left(t\right)$ i.e. susceptible $S\left(t\right)$, exposed $E\left(t\right)$, Infected persons $I\left(t\right)$, hospitalized persons $H\left(t\right)$, and recovered $R\left(t\right)$ where $M\left(t\right)$= $S\left(t\right)+E\left(t\right)+I\left(t\right)+H\left(t\right)+R\left(t\right)$.

**Figure 1:**
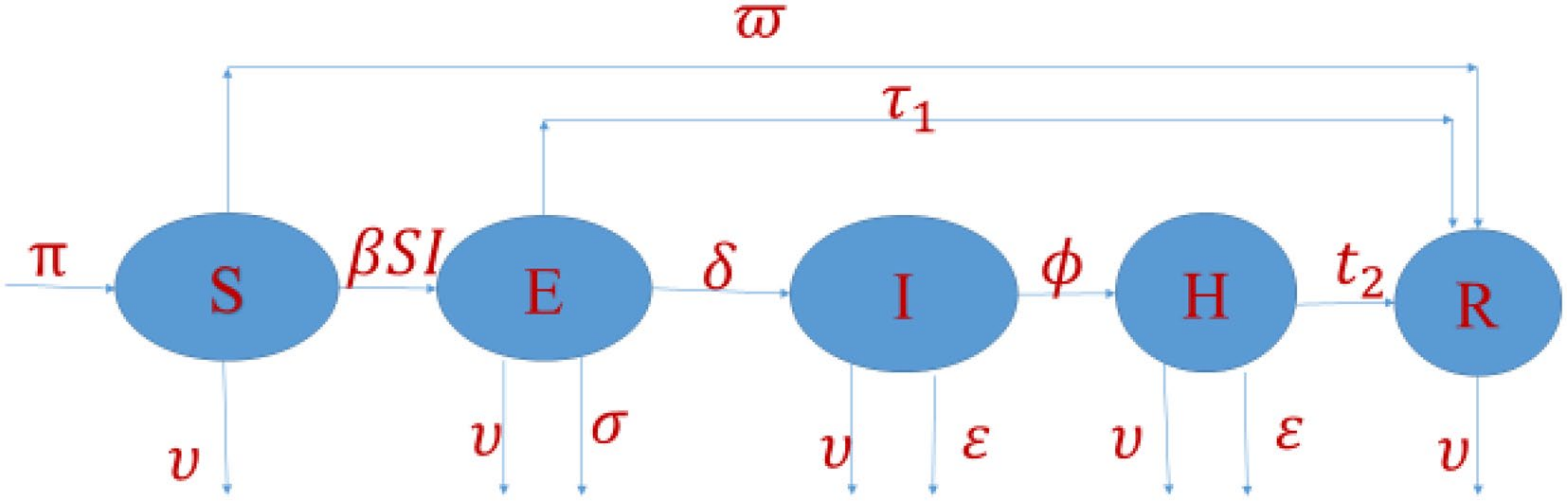
COVID-19 compartmental diagram for mathematical model (1).

From Fig. 1, we can describe the following ${\text{S}},{\text{E}},{\text{I}},{\text{H}},{\text{R}}$ disease model^39^ of differential equations.

1 $$\begin{aligned} & \frac{dS}{{dt}} = {\uppi } - \beta SI - \upsilon S + \varpi R \\ & \frac{dE}{{dt}} = \beta SI - \left( {\delta + \sigma + \upsilon + \tau_{1} } \right)E. \\ & \frac{dI}{{dt}} = \delta E - \left( {\phi + \upsilon + \varepsilon } \right)I. \\ & \frac{dH}{{dt}} = \phi I - \left( {t_{2} + \varepsilon + \upsilon } \right)H \\ & \frac{dR}{{dt}} = \tau_{1} E + t_{2} H - \left( {\varpi + \upsilon } \right)R \\ \end{aligned}$$

### .

The parameters of the proposed COVID-19 model (1) are described in Table 1.

**Table 1:** The parameters and their justifications of the proposed COVID-19 model (1).

Parameters	Description	Numerical value with SI Units
$\pi$	Rate of transfer of people	$0.0000301$ per day
$\beta$	Transmission probability per contact	$0.4995$ per day
${\tau }_{1}$	Early intervention recovery rate	$0.64505$ per day
$\upsilon$	Rate at which those who have recovered are re-infected	$0.00000618$ per day
$\varpi$	Natural death rate of humans	$0.3232$ per day
$\delta$	The portion of hospitalized patients who recover	$0.002981$
$\phi$	Rate of COVID-19-related death	$0.0218$ per day
$\mathcal{E}$	Rate of reinfection among hospitalized COVID-19 patients	$0.38974$ per day
${t}_{2}$	A portion of the population using hand sanitizer and face masks	$0.06813$ per day

## Equilibria and basic reproduction number (${{\varvec{R}}}_{0})$

### Equilibria of the model

The corona-free equilibrium (CFE) point is obtained through setting the equations of model (1) equal to zero. According to this process,${E}_{0}$ = $({S}^{0},{E}^{0},{I}^{0}, {H}^{0},{R}^{0})$ for model (1), then it is easy to find the CFE ${E}_{0}=\left(\frac{\uppi }{\upsilon },\mathrm{0,0},\mathrm{0,0}\right)$. The given model (1) provides an overall solution for the state variables $S$,$E,I,H,$ and $R$ to find the corona endemic equilibrium (CEE) point. If the CEE point is represented by ${E}^{*}\left({S}^{*},{E}^{*},{I}^{*},{H}^{*},{R}^{*}\right)$, then model (1) yields.

$$\frac{\uppi +\varpi {R}^{*}}{(\beta {I}^{*}+\upsilon )}={S}^{*}$$,$${E}^{*}=\frac{\beta {S}^{*}{I}^{*}}{\left(\delta +\sigma +\upsilon +{\tau }_{1}\right)}$$ ,$${I}^{*}=\frac{\delta {E}^{*}}{\left(\phi +\upsilon +\varepsilon \right)}$$ , $${H}^{*}=\frac{\phi {I}^{*}}{\left({t}_{2}+\varepsilon +\upsilon \right)}$$ , and $${R}^{*}=\frac{{\tau }_{1}{E}^{*}+{t}_{2}{H}^{*}}{\left(\varpi +\upsilon \right)}$$.

### Basic reproduction number ${({\varvec{R}}}_{0})$

In our study of COVID-19 transmission dynamics, we utilize the concept of the reproduction number $({R}_{0})$ as an approximation for estimating secondary infections, despite inherent challenges in accurate estimation^41^. By leveraging both the translation and transmission matrices, which capture key disease transmission characteristics, we are able to calculate the basic reproduction number.

$$F\left(x\right)=\left[\begin{array}{c}\beta SI\\ 0\\ 0\end{array}\right],\mathrm{ and }V\left(x\right)=\left[\begin{array}{c}\left(\delta +\sigma +\upsilon +{\tau }_{1}\right)E\\ \left(\phi +\upsilon +\varepsilon \right)I-\delta E\\ \left({t}_{2}+\varepsilon +\upsilon \right)H-\phi I\end{array}\right].$$

As ${R}_{0}=\rho (F{V}^{-1})$, this yields

2 $${R}_{0}=\frac{\beta \delta\uppi }{\upsilon \left(\phi +\upsilon +\varepsilon \right)\left(\delta +\sigma +\upsilon +{\tau }_{1}\right)}$$

## The NSFD scheme

In 1994, Mickens introduced the NSFD concept^43^. The key feature of Mickens' discrete-time epidemic models is that they share the same characteristics as the original continuous-time models. We introduce a numerical NSFD technique for solving Eq. (1), dynamically. The examples below show that the discrete NSFD scheme maintains all the dynamic properties of the equivalent continuous model (1), regardless of the step size (h). The NSFD scheme offers a versatile way to create discrete models and find numerical solutions for both ordinary and partial differential equations. Shokri et al.^42^ state that the effectiveness of the NSFD scheme depends on two factors: estimating nonlinear terms appropriately and discretizing the derivative. Normally, the first-order derivative df/dx is written as $\frac{f\left(y+h\right)-f\left(y\right)}{\varphi (\psi )}$, where $\psi$ represents the step size. According to Mickens^44,45^, this term can be expressed as $\frac{f\left(y+h\right)-f\left(y\right)}{\psi }$, where $\varphi \left(\psi \right)$ is an increasing function known as the denominator function. To better understand the dynamics of COVID-19, we focus on the simplest denominator function, $\varphi \left(\psi \right)=\psi$, in this work rather than a general one as seen in^45^.

The numerical estimates of $S\left(t\right)$,$E\left(t\right)$,$I(t)$,$H\left(t\right)\mathrm{and }R\left(t\right)$ at $t=nh$ for model (1) are denoted as ${S}_{n}, {E}_{n},{I}_{n}, {H}_{n},{R}_{n}$, As well as $n$ being a nonnegative integer, indicates the time for each step. After that, we can write according to model (1).

3 $$\begin{aligned} & \frac{{S_{n + 1} - S_{n} }}{h} = {\uppi } - \beta S_{n + 1} I_{n} - \upsilon S_{n + 1} + \varpi R_{n} \\ & \frac{{E_{n + 1} - E_{n} }}{\psi } = \beta S_{n + 1} I_{n} - \left( {\delta + \sigma + \upsilon + \tau_{1} } \right)E_{n + 1} \\ & \frac{{I_{n + 1} - I_{n} }}{\psi } = \delta E_{n + 1} - \left( {\phi + \upsilon + \varepsilon } \right)I_{n + 1} \\ & \frac{{H_{n + 1} - H_{n} }}{\psi } = \phi I_{n + 1} - \left( {t_{2} + \varepsilon + \upsilon } \right)H_{n + 1} \\ & \frac{{R_{n + 1} - R_{n} }}{\psi } = \tau_{1} E_{n + 1} + t_{2} H_{n + 1} - \left( {\varpi + \upsilon } \right)R_{n + 1} . \\ \end{aligned}$$

The system of the NSFD scheme (3) can be expressed as:

4 $$\begin{aligned} & S_{n + 1} = \frac{{\psi {\uppi } + S_{n} + \psi \varpi R_{n} }}{{\left( {1 + \psi \left( {\beta I_{n} + \upsilon } \right)} \right)}} \\ & E_{n + 1} = \frac{{\psi \beta S_{n + 1} I_{n} + E_{n} }}{{\left( {1 + \psi \left( {\delta + \sigma + \upsilon + \tau_{1} } \right)} \right)}} \\ & I_{n + 1} = \frac{{\psi \delta E_{n + 1} + I_{n} }}{{1 + \psi \left( {\phi + \upsilon + \varepsilon } \right)}} \\ & H_{n + 1} = \frac{{\psi \phi I_{n + 1} + H_{n} }}{{1 + \psi \left( {\left( {t_{2} + \varepsilon + \upsilon } \right)} \right)}} \\ & R_{n + 1} = \frac{{\psi \left( {\tau_{1} E_{n + 1} + t_{2} H_{n + 1} } \right) + R_{n} }}{{1 + \psi \left( {\varpi + \upsilon } \right)}} \\ \end{aligned}$$

### The NSFD scheme's positivity and boundedness

We suppose that the discrete scheme's initial values are positive., i.e. ${S}_{0}\ge 0,{E}_{0}\ge 0,{I}_{0}\ge 0,{H}_{0}\ge 0,{R}_{0}\ge 0$. Because of the expectations, the estimated values for these variables are also nonnegative: ${S}_{n}\ge 0{, E}_{n}\ge 0,{I}_{n}\ge 0,{H}_{n}\ge 0,{R}_{n}\ge 0$. As a result, the NSFD scheme's (4) solutions suggest that the scheme is positive (4), i.e. ${S}_{n+1}\ge 0,{E}_{n+1}\ge 0,{I}_{n+1}\ge 0,{H}_{n+1}\ge 0,{R}_{n+1}\ge 0$. In order to discuss the boundedness of solutions of the NSFD system (4), we consider ${P}_{n}={S}_{n}+{E}_{n}+{I}_{n}+{H}_{n}+{R}_{n}$. Then

$$\frac{{P}_{n+1}-{P}_{n}}{\psi }=\uppi -\upsilon {P}_{n+1},$$

i.e.

$$\left(1+\psi \upsilon \right){P}_{n+1}=\psi\uppi +{P}_{n}.$$

Therefore, we get

$${P}_{n+1}\le \frac{\psi\uppi }{\left(\left(1+\psi \upsilon \right)\right)}+\frac{{P}_{n}}{\left(\left(1+\psi \upsilon \right)\right)}\Leftrightarrow \psi\uppi {\sum }_{k+1}^{n}{\left(\frac{1}{((1+\psi \upsilon )}\right)}^{k}+{P}_{0}{\left(\frac{1}{\left(1+\psi \upsilon \right)}\right)}^{n}.$$

If $0<P\left(0\right)<\frac{\uppi }{\upsilon }$, then by using Gronwall’s inequality, we find

$${P}_{n}\le \frac{\uppi }{\upsilon }\left(1-\frac{1}{{\left(1+\psi \upsilon \right)}^{n}}\right)+{P}_{0}{\left(\frac{1}{1+\psi \upsilon }\right)}^{n}=\frac{\uppi }{\upsilon }+\left({P}_{0}-\frac{\uppi }{\upsilon }\right){\left(\frac{1}{1+\psi \upsilon }\right)}^{n}.$$

Since ${\left(\frac{1}{1+\psi \upsilon }\right)}^{n}<1$, we obtain $P_{n} \to \frac{{\uppi }}{\upsilon }$ as $n \to \infty .$ This demonstrates that the system′s (4) solutions are bounded, and the viable region changes as a result.

$$B=\left\{\left({S}_{n}+{E}_{n}+{I}_{n}+{H}_{n}+{R}_{n}\right):0\le {S}_{n}+{E}_{n}+{I}_{n}+{H}_{n}+{R}_{n}\le \frac{\uppi }{\upsilon }\right\}.$$

Here we can confirm the local stability of equilibrium states for the NSFD scheme (4).

5 $$\begin{aligned} & S_{n + 1} = \frac{{\psi {\uppi } + S_{n} + \varpi R_{n} }}{{\left( {1 + \psi \left( {\beta I_{n} + \upsilon } \right)} \right)}} = l_{1} \\ & E_{n + 1} = \frac{{\psi \beta S_{n + 1} I_{n} + E_{n} }}{{\left( {1 + \psi \left( {\delta + \sigma + \upsilon + \tau_{1} } \right)} \right)}} = l_{2} \\ & I_{n + 1} = \frac{{\psi \delta E_{n + 1} + I_{n} }}{{1 + \psi \left( {\phi + \upsilon + \varepsilon } \right)}} = l_{3} \\ & H_{n + 1} = \frac{{\psi \phi I_{n + 1} + H_{n} }}{{1 + \psi \left( {\left( {t_{2} + \varepsilon + \upsilon } \right)} \right)}} = l_{4} \\ & R_{n + 1} = \frac{{\psi \left( {\tau_{1} E_{n + 1} + t_{2} H_{n + 1} } \right) + R_{n} }}{{1 + \psi \left( {\varpi + \upsilon } \right)}} = l_{5} . \\ \end{aligned}$$

### Local stability of equilibria

In order to demonstrate that the CFE point is locally asymptotically stable (LAS), we will apply the Schur-Cohn criterion^46,49^ as defined in the following Lemma 1.

#### Lemma 1

The roots of ${\mathrm{\rm T}}^{2}-D\mathrm{\rm T}+E=0$ guarantee $\left|{\mathrm{\rm T}}_{k}\right|<1 \mathrm{for } k=\mathrm{1,2}$, $\iff$ the following requirements are satisfied.

1. $$E<1$$,
2. $$1+D+E>0$$,
3. $$1-D+E>0$$,

where $D$ describes trace and the $E$ mentioned is the determinant of the Jacobian matrix.

#### Theorem 1

If $\psi >0$, the CFE is LAS for NSFD model (4) when ${R}_{0}<1$.

#### Proof

The Jacobian matrix can be expressed as the following using the information presented above.

6 $$J\left(S,E,I,H,R\right)=\left[\begin{array}{ccccc}\frac{\partial {l}_{1}}{\partial S}& \frac{\partial {l}_{1}}{\partial E}& \frac{\partial {l}_{1}}{\partial I}& \frac{\partial {l}_{1}}{\partial H}& \frac{\partial {l}_{1}}{\partial R}\\ \frac{\partial {l}_{2}}{\partial S}& \frac{\partial {l}_{2}}{\partial E}& \frac{\partial {l}_{2}}{\partial I}& \frac{\partial {l}_{2}}{\partial H}& \frac{\partial {l}_{2}}{\partial R}\\ \frac{\partial {l}_{3}}{\partial S}& \frac{\partial {l}_{3}}{\partial E}& \frac{\partial {l}_{3}}{\partial I}& \frac{\partial {l}_{3}}{\partial H}& \frac{\partial {l}_{3}}{\partial R}\\ \frac{\partial {l}_{4}}{\partial S}& \frac{\partial {l}_{4}}{\partial E}& \frac{\partial {l}_{4}}{\partial I}& \frac{\partial {l}_{4}}{\partial H}& \frac{\partial {l}_{4}}{\partial R}\\ \frac{\partial {l}_{5}}{\partial S}& \frac{\partial {l}_{5}}{\partial E}& \frac{\partial {l}_{5}}{\partial I}& \frac{\partial {l}_{5}}{\partial H}& \frac{\partial {l}_{5}}{\partial R}\end{array}\right],$$

where ${l}_{1},{l}_{2},{l}_{3},{l}_{4}$ and ${l}_{5}$ are provided in (5), a list of derivatives that can be found in (6) is as follows:

$$\begin{aligned} & \frac{{\partial l_{1} }}{\partial S} = \frac{1}{{\left( {1 + \psi \left( {\beta I_{n} + \upsilon } \right)} \right)}},\frac{{\partial l_{1} }}{\partial E} = 0,\frac{{\partial l_{1} }}{\partial I} = \frac{{ - \psi {\uppi }}}{{\left( {1 + \psi \left( {\beta I_{n} + \upsilon } \right)} \right)^{2} }} ,\frac{{\partial l_{1} }}{\partial H} = 0,\frac{{\partial l_{1} }}{\partial R} = \frac{\varpi }{{\left( {1 + \psi \left( {\beta I_{n} + \upsilon } \right)} \right)}}, \\ & \frac{{\partial l_{2} }}{\partial S} = \frac{{\psi \beta S_{n + 1} I_{n} }}{{\left( {1 + \psi \left( {\delta + \sigma + \upsilon + \tau_{1} } \right)} \right)}}\frac{{\partial l_{2} }}{\partial E} = \frac{1}{{\left( {1 + \psi \left( {\delta + \sigma + \upsilon + \tau_{1} } \right)} \right)}}\frac{{\partial l_{2} }}{\partial I} = \frac{{\psi \beta S_{n + 1} }}{{\left( {1 + \psi \left( {\delta + \sigma + \upsilon + \tau_{1} } \right)} \right)}}\frac{{\partial l_{2} }}{\partial H} = 0\frac{{\partial l_{2} }}{\partial R} = 0, \\ & \frac{{\partial l_{3} }}{\partial S} = 0\frac{{\partial l_{3} }}{\partial E} = \frac{\psi \delta }{{1 + \psi \left( {\phi + \upsilon + \varepsilon } \right)}}\frac{{\partial l_{3} }}{\partial I} = \frac{1}{{1 + \psi \left( {\phi + \upsilon + \varepsilon } \right)}}\frac{{\partial l_{3} }}{\partial H} = 0\frac{{\partial l_{3} }}{\partial R} = 0,\frac{{\partial l_{4} }}{\partial S} = 0, \frac{{\partial l_{4} }}{\partial E} = 0 , \frac{{\partial l_{4} }}{\partial I} = \frac{h\phi }{{1 + \psi \left( {\left( {t_{2} + \varepsilon + \upsilon } \right)} \right)}}, \\ & \frac{{\partial l_{4} }}{\partial H} = \frac{1}{{1 + \psi \left( {\left( {t_{2} + \varepsilon + \upsilon } \right)} \right)}}, \frac{{\partial l_{4} }}{\partial R} = 0,\frac{{\partial l_{5} }}{\partial S} = 0, \frac{{\partial l_{5} }}{\partial E} = \frac{{h\tau_{1} }}{{1 + \psi \left( {\varpi + \upsilon } \right)}}, \frac{{\partial l_{5} }}{\partial I} = 0, \frac{{\partial l_{5} }}{\partial H} = \frac{{h\tau_{2} }}{{1 + \psi \left( {\varpi + \upsilon } \right)}}, \frac{{\partial l_{5} }}{\partial R} = \frac{1}{{1 + \psi \left( {\varpi + \upsilon } \right)}}, \\ \end{aligned}$$

substituting all the above derivatives in (6), we get

7 $$J=\left[\begin{array}{ccccc}\frac{1}{\left(1+\psi \left(\beta {I}_{n}+\upsilon \right)\right)}& 0& \frac{-\psi\uppi }{{\left(1+\psi \left(\beta {I}_{n}+\upsilon \right)\right)}^{2}}& 0& \frac{\varpi }{\left(1+\psi \left(\beta {I}_{n}+\upsilon \right)\right)}\\ \frac{\psi \beta {I}_{n}}{\left(1+\psi \left(\delta +\sigma +\upsilon +{\tau }_{1}\right)\right)}& \frac{1}{\left(1+\psi \left(\delta +\sigma +\upsilon +{\tau }_{1}\right)\right)}& \frac{\psi \beta {S}_{n+1}}{\left(1+\psi \left(\delta +\sigma +\upsilon +{\tau }_{1}\right)\right)}& 0& 0\\ 0& \frac{\psi \delta }{1+\psi \left(\phi +\upsilon +\varepsilon \right)}& \frac{1}{1+\psi \left(\phi +\upsilon +\varepsilon \right)}& 0& 0\\ 0& 0& \frac{\psi \phi }{1+\psi \left(\left({t}_{2}+\varepsilon +\upsilon \right)\right)}& \frac{1}{1+\psi \left(\left({t}_{2}+\varepsilon +\upsilon \right)\right)}& 0\\ 0& \frac{\psi {\tau }_{1}}{1+\psi \left(\varpi +\upsilon \right)}& 0& \frac{\psi {\tau }_{2}}{1+\psi \left(\varpi +\upsilon \right)}& \frac{1}{1+\psi \left(\varpi +\upsilon \right)}\end{array}\right].$$

At CFE point ${E}_{0}=\left(\frac{\uppi }{\upsilon },\mathrm{0,0},\mathrm{0,0}\right)$, the matrix (7) becomes

$$J\left({E}_{0}\right)=\left[\begin{array}{ccccc}\frac{1}{\left(1+\psi \upsilon \right)}& 0& \frac{-\psi\uppi }{{\left(1+\psi \upsilon \right)}^{2}}& 0& \frac{\varpi }{\left(1+h\upsilon \right)}\\ 0& \frac{1}{\left(1+\psi \left(\delta +\sigma +\upsilon +{\tau }_{1}\right)\right)}& \frac{\psi \beta\uppi }{\upsilon \left(1+\psi \left(\delta +\sigma +\upsilon +{\tau }_{1}\right)\right)}& 0& 0\\ 0& \frac{\psi \delta }{1+\psi \left(\phi +\upsilon +\varepsilon \right)}& \frac{1}{1+\psi \left(\phi +\upsilon +\varepsilon \right)}& 0& 0\\ 0& 0& \frac{\psi \phi }{1+\psi \left(\left({t}_{2}+\varepsilon +\upsilon \right)\right)}& \frac{1}{1+\psi \left(\left({t}_{2}+\varepsilon +\upsilon \right)\right)}& 0\\ 0& \frac{\psi {\tau }_{1}}{1+\psi \left(\varpi +\upsilon \right)}& 0& \frac{h{\tau }_{2}}{1+\psi \left(\varpi +\upsilon \right)}& \frac{1}{1+\psi \left(\varpi +\upsilon \right)}\end{array}\right].$$

To find the eigenvalues, we solve.

$$\left|J\left({E}_{0}\right)-I\right|=0,$$

i.e.

8 $$\left|\begin{array}{ccccc}\frac{1}{\left(1+\psi \upsilon \right)}-\mathrm{\rm T}& 0& \frac{-\psi\uppi }{{\left(1+\psi \upsilon \right)}^{2}}& 0& \frac{\varpi }{\left(1+\psi \upsilon \right)}\\ 0& \frac{1}{\left(1+\psi \left(\delta +\sigma +\upsilon +{\tau }_{1}\right)\right)}-\mathrm{\rm T}& \frac{h\beta\uppi }{\upsilon \left(1+\psi \left(\delta +\sigma +\upsilon +{\tau }_{1}\right)\right)}& 0& 0\\ 0& \frac{\psi \delta }{1+\psi \left(\phi +\upsilon +\varepsilon \right)}& \frac{1}{1+\psi \left(\phi +\upsilon +\varepsilon \right)}-\mathrm{\rm T}& 0& 0\\ 0& 0& \frac{h\phi }{1+\psi \left(\left({t}_{2}+\varepsilon +\upsilon \right)\right)}& \frac{1}{1+\psi \left(\left({t}_{2}+\varepsilon +\upsilon \right)\right)}-\mathrm{\rm T}& 0\\ 0& \frac{\psi {\tau }_{1}}{1+\psi \left(\varpi +\upsilon \right)}& 0& \frac{h{\tau }_{2}}{1+\psi \left(\varpi +\upsilon \right)}& \frac{1}{1+\psi \left(\varpi +\upsilon \right)}-\mathrm{\rm T}\end{array}\right|=0.$$

On simplifying, (8) yields

9 $$\left(\frac{1}{\left(1+\psi \upsilon \right)}-{\mathrm{\rm T}}_{1}\right)\left(\frac{1}{1+\psi \left(\varpi +\upsilon \right)}-{\mathrm{\rm T}}_{2}\right)\left(\frac{1}{1+\psi \left(\left({t}_{2}+\varepsilon +\upsilon \right)\right)}-{\mathrm{\rm T}}_{3}\right)\left|\begin{array}{cc}\frac{1}{\left(1+\psi \left(\delta +\sigma +\upsilon +{\tau }_{1}\right)\right)}-\mathrm{\rm T}& \frac{\psi \beta\uppi }{\upsilon \left(1+\psi \left(\delta +\sigma +\upsilon +{\tau }_{1}\right)\right)}\\ \frac{\psi \delta }{1+\psi \left(\phi +\upsilon +\varepsilon \right)}& \frac{1}{1+\psi \left(\phi +\upsilon +\varepsilon \right)}-\mathrm{\rm T}\end{array}\right|=0.$$

The Eq. (9) provides ${\mathrm{\rm T}}_{1}=\frac{1}{\left(1+\psi \upsilon \right)}<1,{T}_{2}=\frac{1}{1+\psi \left(\varpi +\upsilon \right)}<1$ and ${T}_{3}=\frac{1}{1+\psi \left(\left({t}_{2}+\varepsilon +\upsilon \right)\right)}<1$.

To find other eigenvalues, we take

$$\left|\begin{array}{cc}\frac{1}{\left(1+\psi \left(\delta +\sigma +\upsilon +{\tau }_{1}\right)\right)}-T& \frac{\psi \beta\uppi }{\upsilon \left(1+\psi \left(\delta +\sigma +\upsilon +{\tau }_{1}\right)\right)}\\ \frac{\psi \delta }{1+\psi ``\left(\phi +\upsilon +\varepsilon \right)}& \frac{1}{1+\psi \left(\phi +\upsilon +\varepsilon \right)}-T\end{array}\right|=0,$$

i.e.

10 $${T}^{2}-T\left(\frac{1}{\left(1+\psi \left(\delta +\sigma +\upsilon +{\tau }_{1}\right)\right)}+\frac{1}{1+\psi \left(\phi +\upsilon +\varepsilon \right)}\right)+\frac{1}{\left(1+\psi \left(\delta +\sigma +\upsilon +{\tau }_{1}\right)\right)}\frac{1}{1+\psi \left(\phi +\upsilon +\varepsilon \right)}-\frac{\psi \delta }{1+\psi ``\left(\phi +\upsilon +\varepsilon \right)}\frac{\psi \beta\uppi }{\upsilon \left(1+\psi \left(\delta +\sigma +\upsilon +{\tau }_{1}\right)\right)}=0.$$

1. $$E<1$$,
2. $$1+D+E>0$$,
3. $$1-D+E>0$$,

Comparing Eq. (10) with ${T}^{2}-DT+E=0$, we get $D=\left(\frac{1}{\left(1+\psi \left(\delta +\sigma +\upsilon +{\tau }_{1}\right)\right)}+\frac{1}{1+\psi \left(\phi +\upsilon +\varepsilon \right)}\right)$ and $E=\frac{1}{\left(1+\psi \left(\delta +\sigma +\upsilon +{\tau }_{1}\right)\right)}\frac{1}{1+\psi \left(\phi +\upsilon +\varepsilon \right)}-\frac{\psi \delta }{1+\psi ``\left(\phi +\upsilon +\varepsilon \right)}\frac{\psi \beta\uppi }{\upsilon \left(1+\psi \left(\delta +\sigma +\upsilon +{\tau }_{1}\right)\right)}$. If ${R}_{0}<1,$

1. $$E=\frac{1}{\left(1+\psi \left(\delta +\sigma +\upsilon +{\tau }_{1}\right)\right)}+\frac{1}{1+\psi \left(\phi +\upsilon +\varepsilon \right)}<1$$.
2. $$1+D+E=1+\frac{1}{\left(1+\psi \left(\delta +\sigma +\upsilon +{\tau }_{1}\right)\right)}+\frac{1}{1+\psi \left(\phi +\upsilon +\varepsilon \right)}+\frac{1}{\left(1+\psi \left(\delta +\sigma +\upsilon +{\tau }_{1}\right)\right)}\frac{1}{1+\psi \left(\phi +\upsilon +\varepsilon \right)}-\frac{\psi \delta }{1+\psi \left(\phi +\upsilon +\varepsilon \right)}\frac{\psi \beta\uppi }{\upsilon \left(1+\psi \left(\delta +\sigma +\upsilon +{\tau }_{1}\right)\right)}>0$$.
3. $$1-D+E=1-\frac{1}{\left(1+\psi \left(\delta +\sigma +\upsilon +{\tau }_{1}\right)\right)}+\frac{1}{1+\psi \left(\phi +\upsilon +\varepsilon \right)}+\frac{1}{\left(1+\psi \left(\delta +\sigma +\upsilon +{\tau }_{1}\right)\right)}\frac{1}{1+\psi \left(\phi +\upsilon +\varepsilon \right)}-\frac{\psi \delta }{1+\psi \left(\phi +\upsilon +\varepsilon \right)}\frac{\psi \beta\uppi }{\upsilon \left(1+\psi \left(\delta +\sigma +\upsilon +{\tau }_{1}\right)\right)}>0$$.

In Eq. (2) when we put the numerical values of all positive parameter, its gives us a value greater than zero. So, we can say that the Eq. (2) is greater than zero.

The Schur-Cohn condition is therefore met whenever ${R}_{0}<1$, according to Lemma 1. The CFE point ${E}_{0}$ of the discrete NSFD scheme (4) therefore becomes locally asymptotically stable when ${R}_{0}<1$, is satisfied.

In order to talk about CEE point LAS, replace ${R}_{n}$ by $\left(\frac{\uppi }{\upsilon }-{S}_{n}-{E}_{n}-{I}_{n}-{H}_{n}\right)$ in the first equation of system (3). Then, obviously the system (3) reduces to

5 $$\begin{aligned} & \frac{{S_{n + 1} - S_{n} }}{\psi } = {\uppi } - \beta S_{n + 1} I_{n} - \upsilon S_{n + 1} + \varpi \left( {\frac{{\uppi }}{\upsilon } - S_{n} - E_{n} - I_{n} - H_{n} } \right) \\ & \frac{{E_{n + 1} - E_{n} }}{\psi } = \beta S_{n + 1} I_{n} - \left( {\delta + \sigma + \upsilon + \tau_{1} } \right)E_{n + 1} \\ & \frac{{I_{n + 1} - I_{n} }}{\psi } = \delta E_{n + 1} - \left( {\phi + \upsilon + \varepsilon } \right)I_{n + 1} \\ & \frac{{H_{n + 1} - H_{n} }}{\psi } = \phi I_{n + 1} - \left( {t_{2} + \varepsilon + \upsilon } \right)H_{n + 1} \\ \end{aligned}$$

Now the system becomes

$$S_{n + 1} = \frac{{\psi {\uppi } + S_{n} + \varpi \left( {\frac{{\uppi }}{\upsilon } - S_{n} - E_{n} - I_{n} - H_{n} } \right)}}{{\left( {1 + \psi \left( {\beta I_{n} + \upsilon } \right)} \right)}} = k_{1}$$

$$E_{n + 1} = \frac{{h\beta S_{n + 1} I_{n} + E_{n} }}{{\left( {1 + h\left( {\delta + \sigma + \upsilon + \tau_{1} } \right)} \right)}} = k_{2}$$

5 $$I_{n + 1} = \frac{{\psi \delta E_{n + 1} + I_{n} }}{{1 + \psi \left( {\phi + \upsilon + \varepsilon } \right)}} = k_{3}$$

$$H_{n + 1} = \frac{{\psi \phi I_{n + 1} + H_{n} }}{{1 + \psi \left( {\left( {t_{2} + \varepsilon + \upsilon } \right)} \right)}} = k_{4}$$

where ${k}_{1},{k}_{2},{k}_{3 }\mathrm{and }{k}_{4}$ are provided in system (5). First we find derivatives employed that

$$\frac{\partial {k}_{1}}{\partial S}=\frac{1}{\left(1+\psi \left(\beta {I}_{n}+\upsilon \right)\right)},\frac{\partial {k}_{1}}{\partial E}=0,\frac{\partial {k}_{1}}{\partial I}=\frac{-\psi\uppi }{{\left(1+\psi \left(\beta {I}_{n}+\upsilon \right)\right)}^{2}} ,\frac{\partial {k}_{1}}{\partial H}=0,$$

$$\frac{\partial {k}_{2}}{\partial S}=\frac{\psi \beta {S}_{n+1}{I}_{n}}{\left(1+\psi \left(\delta +\sigma +\upsilon +{\tau }_{1}\right)\right)},\frac{\partial {k}_{2}}{\partial E}=\frac{1}{\left(1+\psi \left(\delta +\sigma +\upsilon +{\tau }_{1}\right)\right)},\frac{\partial {k}_{2}}{\partial I}=\frac{\psi \beta {S}_{n+1}}{\left(1+\psi \left(\delta +\sigma +\upsilon +{\tau }_{1}\right)\right)},\frac{\partial {l}_{2}}{\partial H}=0,\frac{\partial {k}_{3}}{\partial S}=0,\frac{\partial {k}_{3}}{\partial E}=\frac{\psi \delta }{1+\psi \left(\phi +\upsilon +\varepsilon \right)},\frac{\partial {k}_{3}}{\partial I}=\frac{1}{1+\psi \left(\phi +\upsilon +\varepsilon \right)},$$

$$\frac{\partial {k}_{3}}{\partial H}=0,\frac{\partial {k}_{4}}{\partial S}=0, \frac{\partial {k}_{4}}{\partial E}=0 , \frac{\partial {k}_{4}}{\partial I}=\frac{\psi \phi }{1+\psi \left(\left({t}_{2}+\varepsilon +\upsilon \right)\right)}, \frac{\partial {k}_{4}}{\partial H}=\frac{1}{1+\psi \left(\left({t}_{2}+\varepsilon +\upsilon \right)\right)},$$

#### Theorem 2

If ${R}_{0}>1$, then CEE point for NSFD model (4) is LAS for all $\psi >0$.

#### Proof

In the similar way to Theorem 1, we get the Jacobian matrix.

11 $$J=\left[\begin{array}{cccc}\frac{1}{\left(1+\psi \left(\beta {I}_{n}+\upsilon \right)\right)}& 0& \frac{-\psi\uppi }{{\left(1+\psi \left(\beta {I}_{n}+\upsilon \right)\right)}^{2}}& 0\\ \frac{h\beta {I}_{n}}{\left(1+\psi \left(\delta +\sigma +\upsilon +{\tau }_{1}\right)\right)}& \frac{1}{\left(1+\psi \left(\delta +\sigma +\upsilon +{\tau }_{1}\right)\right)}& \frac{h\beta {S}_{n+1}}{\left(1+\psi \left(\delta +\sigma +\upsilon +{\tau }_{1}\right)\right)}& 0\\ 0& \frac{h\delta }{1+\psi \left(\phi +\upsilon +\varepsilon \right)}& \frac{1}{1+\psi \left(\phi +\upsilon +\varepsilon \right)}& 0\\ 0& 0& \frac{h\phi }{1+\psi \left(\left({t}_{2}+\varepsilon +\upsilon \right)\right)}& \frac{1}{1+\psi \left(\left({t}_{2}+\varepsilon +\upsilon \right)\right)}\end{array}\right].$$

By putting CEE point ${E}^{*}$ in (11), we get

12 $$J\left({E}^{*}\right)=\left[\begin{array}{cccc}\frac{1}{\left(1+\psi \left(\beta {I}^{*}+\upsilon \right)\right)}& 0& \frac{-h\uppi }{{\left(1+\psi \left(\beta {I}^{*}+\upsilon \right)\right)}^{2}}& 0\\ \frac{\psi \beta {I}^{*}}{\left(1+\psi \left(\delta +\sigma +\upsilon +{\tau }_{1}\right)\right)}& \frac{1}{\left(1+\psi \left(\delta +\sigma +\upsilon +{\tau }_{1}\right)\right)}& \frac{h\beta {S}^{*}}{\left(1+\psi \left(\delta +\sigma +\upsilon +{\tau }_{1}\right)\right)}& 0\\ 0& \frac{h\delta }{1+\psi \left(\phi +\upsilon +\varepsilon \right)}& \frac{1}{1+\psi \left(\phi +\upsilon +\varepsilon \right)}& 0\\ 0& 0& \frac{h\phi }{1+\psi \left(\left({t}_{2}+\varepsilon +\upsilon \right)\right)}& \frac{1}{1+\psi \left(\left({t}_{2}+\varepsilon +\upsilon \right)\right)}\end{array}\right].$$

Let

$${m}_{1}=-\frac{1}{\left(1+\psi \left(\beta {I}^{*}+\upsilon \right)\right)},{m}_{2}=\frac{-\psi\uppi }{{\left(1+\psi \left(\beta {I}^{*}+\upsilon \right)\right)}^{2}},{m}_{3}=-\frac{\varpi }{\left(1+\psi \left(\beta {I}^{*}+\upsilon \right)\right)},{m}_{4}=-\frac{\psi \beta {I}^{*}}{\left(1+\psi \left(\delta +\sigma +\upsilon +{\tau }_{1}\right)\right)},$$

$${m}_{5}=-\frac{1}{\left(1+\psi \left(\delta +\sigma +\upsilon +{\tau }_{1}\right)\right)},{m}_{6}=-\frac{\psi \beta {S}^{*}}{\left(1+\psi \left(\delta +\sigma +\upsilon +{\tau }_{1}\right)\right)},{m}_{7}=-\frac{\psi \delta }{1+\psi \left(\phi +\upsilon +\varepsilon \right)},{m}_{8}=-\frac{1}{1+\psi \left(\phi +\upsilon +\varepsilon \right)},{m}_{9}=-\frac{\psi \phi }{1+\psi \left(\left({t}_{2}+\varepsilon +\upsilon \right)\right)}$$.

By replacing the above quantities in (12), we obtain

$$J\left({E}^{*}\right)=\left[\begin{array}{cccc}{m}_{1}& 0& {-m}_{2}& 0\\ {m}_{4}& {m}_{5}& {m}_{6}& 0\\ 0& {m}_{7}& {m}_{8}& 0\\ 0& 0& {m}_{9}& {m}_{10}\end{array}\right].$$

To discuss the eigenvalues, we take

$$\left|J\left({E}^{*}\right)-\lambda \right|=0,$$

i.e.

$$\left|\begin{array}{cccc}{m}_{1}-\lambda & 0& {-m}_{2}& 0\\ {m}_{4}& {m}_{5}-\lambda & {m}_{6}& 0\\ 0& {m}_{7}& {m}_{8}-\lambda & 0\\ 0& 0& {m}_{9}& {m}_{10}-\lambda \end{array}\right|=0.$$

$$\left({m}_{10}-\lambda \right)\left[{\lambda }^{3}+{U}_{3}{\lambda }^{2}+{U}_{4}\lambda +U\right]=0.$$

The first Eigen value is $\lambda ={m}_{10}$ and other values are found by solving the characteristic equation,

$$\left[{\lambda }^{3}+{U}_{3}{\lambda }^{2}+{U}_{2}\lambda +{U}_{1}\right]=0.$$

This yields;

$${U}_{3}={m}_{8}+{m}_{5}+{m}_{1}>0.$$

$${U}_{2}={m}_{1}{m}_{5}+{m}_{1}{m}_{8}+{m}_{5}{m}_{8}+{m}_{6}{m}_{7}>0.$$

$${U}_{1}={m}_{8}{m}_{5}{m}_{1}+{m}_{2}{m}_{4}{m}_{7}-{m}_{7}{m}_{6}{m}_{1}>0.$$

$${U}_{1}{U}_{2}-{U}_{3}=\left({m}_{8}{m}_{5}{m}_{1}+{m}_{2}{m}_{4}{m}_{7}-{m}_{7}{m}_{6}{m}_{1}\right)\left({m}_{1}{m}_{5}+{m}_{1}{m}_{8}+{m}_{5}{m}_{8}+{m}_{6}{m}_{7}\right)-{m}_{8}+{m}_{5}+{m}_{1}>0.$$

All the values are positive, this is numerically proven, we can thus say that ${U}_{1},{U}_{2},{U}_{3}>0$.

Thus, the Routh-Hurwitz criterion^47,48^ is satisfied. So, CEE point ${E}^{*}$ of system (4.2) is LAS.

### Global stability of equilibria

To find the global stability of CFE and CEE points for NSFD scheme (4), we describe the function $N(x)\ge 0$ such that $H\left(x\right)=Z-{\text{ln}}Z-1$ and, so ${\text{ln}}Z\le Z-1.$

#### Theorem 3

For all $\psi >0$, the CFE point is globally asymptotically stable (GAS) for NSFD model (4) whenever ${R}_{0}\le 1$.

#### Proof

Create a discrete Lyapunov function.

$${X}_{n}\left({S}_{n}{, E}_{n},{I}_{n},{H}_{n},{R}_{n}\right)={S}^{0}N\left(\frac{{S}_{n}}{{S}^{0}}\right)+{\varphi }_{1}{E}_{n}+{\varphi }_{2}{I}_{n}+{\varphi }_{3}{H}_{n}+{\varphi }_{4}{R}_{n},$$

where ${\varphi }_{j}>0$ for all $j=\mathrm{1,2},\mathrm{3,4}$. Hence, ${X}_{n}>0$ for all ${S}_{n}>0, {E}_{n}>0, {I}_{n}>0, {{H}_{n}>0, \mathrm{and } R}_{n}>0$. In addition, ${X}_{n}=0,$ if and only if ${S}_{n}={S}^{0},{E}_{n}={E}^{0}, {I}_{n}={I}^{0},{H}_{n}={H}^{0},$ and ${R}_{n}$=

$${R}^{0}$$. We take

$$\Delta {X}_{n}={X}_{n+1}-{X}_{n},$$

**Figure 2:**
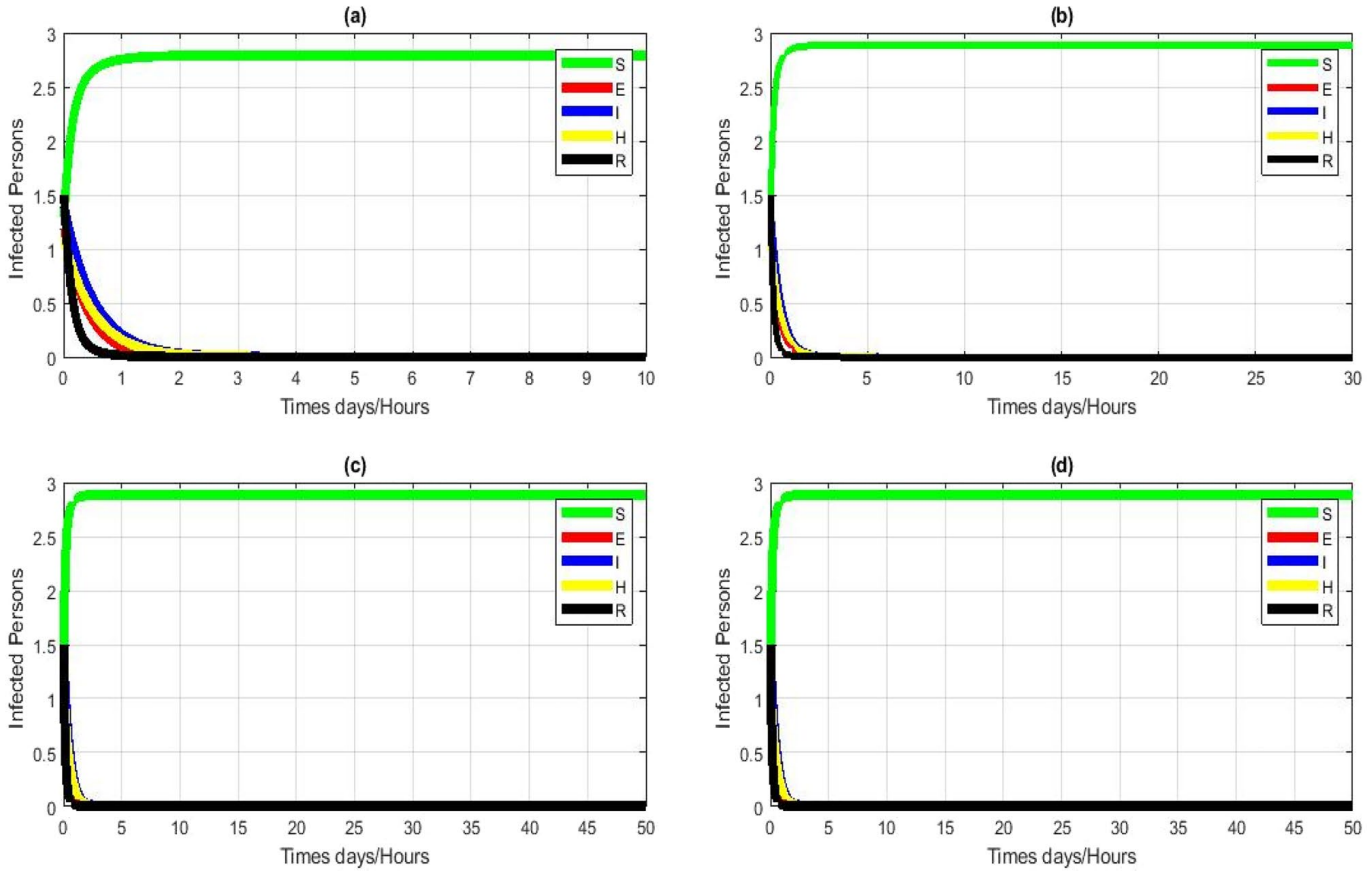
Numerical simulation for model (1) by using NSFD scheme with $\left(\mathbf{a}\right) h = 0.01,\left(\mathbf{b}\right) h = 0.1,\left(\mathbf{c}\right) h=1,\left(\mathbf{d}\right) h=10$. (**a**–**d**) Stable CFE point with $\uppi =0.0000301$ other parameters remain fixed $\beta =0.00000618$,${\tau }_{1}=0.64505$,$\theta =0.02189 \upsilon =0.4995$, $\varpi =0.3232$, $\delta =0.002981,\mathcal{E}=0.38974$, ${t}_{2}=0.06813$.

The numerical simulations shown in Fig. 2a–d for ${R}_{0}<1$ respectively also exhibit that the solutions of NSFD scheme (4) converges to the CFE point ${E}_{0}$ independent of the step size. Time-independent solutions for the system (1) using different initial conditions. Each color represents a solution.

i.e.

15 $$\begin{aligned} \Delta X_{n} & = S^{0} F\left( {\frac{{S_{n + 1} }}{{S^{0} }}} \right) + \varphi_{1} E_{n + 1} + \varphi_{2} I_{n + 1} + \varphi_{3} H_{n + 1} + \varphi_{4} R_{n + 1} - \left( {S^{0} F\left( {\frac{{S_{n} }}{{S^{0} }}} \right) + \varphi_{1} E_{n} + \varphi_{3} I_{n} + \varphi_{4} H_{n} + \varphi_{3} R_{n} } \right). \\ & = S^{0} \left( {\frac{{S_{n + 1} }}{{S^{0} }} - \frac{{S_{n} }}{{S^{0} }} + \ln \frac{{S_{n } }}{{S_{n + 1} }}} \right) + \varphi_{1} \left( {E_{n + 1} - E_{n} } \right) + \varphi_{2} \left( {I_{n + 1} - I_{n} } \right) + \varphi_{3} \left( {H_{n + 1} - H_{n} } \right) + \varphi_{4} \left( {R_{n + 1} - R_{n} } \right) \\ \end{aligned}$$

Using the inequality ${\text{ln}}Z\le Z-1$, (15) becomes

16 $$\begin{aligned} \Delta X_{n} & \le S_{n + 1} - S_{n} + S^{0} \left( { - 1 + \frac{{S_{n } }}{{S_{n + 1} }}} \right) + \left( { - 1 + \frac{{E_{n } }}{{E_{n + 1} }}} \right)\varphi_{1} \left( {E_{n + 1} - E_{n} } \right) + \left( { - 1 + \frac{{I_{n } }}{{I_{n + 1} }}} \right)\varphi_{2} \left( {I_{n + 1} - I_{n} } \right) + \left( { - 1 + \frac{{H_{n} }}{{H_{n + 1} }}} \right)\varphi_{3} \left( {H_{n + 1} - H_{n} } \right) + \left( { - 1 + \frac{{R_{n} }}{{R_{n + 1} }}} \right)\varphi_{4} \left( {R_{n + 1} - R_{n} } \right). \\ & = - \left( {1 - \frac{{S^{0} }}{{S_{n + 1} }}} \right)\left( {S_{n + 1} - S_{n} } \right) - \left( {1 - \frac{{E_{n } }}{{E_{n + 1} }}} \right)\varphi_{1} \left( {E_{n + 1} n - E_{n} } \right) - \left( {1 - \frac{{I_{n } }}{{I_{n + 1} }}} \right)\varphi_{2} \left( {I_{n + 1} - I_{n} } \right) - \left( {1 - \frac{{H_{n} }}{{H_{n + 1} }}} \right)\varphi_{3} \left( {H_{n + 1} - H_{n} } \right) - \left( {1 - \frac{{R_{n} }}{{R_{n + 1} }}} \right)\varphi_{4} \left( {R_{n + 1} - R_{n} } \right). \\ \end{aligned}$$

Using system (3), (16) can be expressed as

17 $$\begin{aligned} \Delta X_{n} & \le - \left( {\left( {1 - \frac{{S^{0} }}{{S_{n + 1} }}} \right)\left( {{\uppi } - \beta S_{n + 1} I_{n} - \upsilon S_{n + 1} + \varpi R_{n} } \right) + \left( {1 - \frac{{E_{n } }}{{E_{n + 1} }}} \right)\phi_{1} \left( {\beta S_{n + 1} I_{n} - \left( {\delta + \sigma + \upsilon + \tau_{1} } \right)E_{n + 1} } \right)} \right. \\ & \quad + \left( {1 - \frac{{I_{n } }}{{I_{n + 1} }}} \right)\phi_{2} \left( {\delta E_{n + 1} - \left( {\phi + \upsilon + \varepsilon } \right)I_{n + 1} } \right) + \left( {1 - \frac{{H_{n} }}{{H_{n + 1} }}} \right)\phi_{3} \left( {\phi I_{n + 1} - \left( {t_{2} + \varepsilon + \upsilon } \right)H_{n + 1} } \right) \\ & \quad \left. { + \left( {1 - \frac{{R_{n} }}{{R_{n + 1} }}} \right)\phi_{4} \left( {\tau_{1} E_{n + 1} + t_{2} H_{n + 1} - \left( {\varpi + \upsilon } \right)R_{n + 1} } \right)} \right). \\ \end{aligned}$$

Let ${\varphi }_{j}$ for $j=\mathrm{1,2},\mathrm{3,4}$ be nominated so that

$$\begin{aligned} & \left( {{\uppi } - \beta S_{n + 1} I_{n} - \upsilon S_{n + 1} + \varpi R_{n} } \right) = \varphi_{1} \left( {\beta S_{n + 1} I_{n} - \left( {\delta + \sigma + \upsilon + \tau_{1} } \right)E_{n + 1} } \right), \\ & \varphi_{2} \left( {\delta E_{n + 1} - \left( {\phi + \upsilon + \varepsilon } \right)I_{n + 1} } \right) = \varphi_{3} \left( {\phi I_{n + 1} - \left( {t_{2} + \varepsilon + \upsilon } \right)H_{n + 1} } \right), \\ & \left( {\varphi I_{n + 1} - \left( {t_{2} + \varepsilon + \upsilon } \right)H_{n + 1} } \right) = \varphi_{5} \left( {\tau_{1} E_{n + 1} + t_{2} H_{n + 1} - \left( {\varpi + \upsilon } \right)R_{n + 1} } \right). \\ \end{aligned}$$

By putting the above values, from (17) we get

$$\begin{aligned} \Delta X_{n} & \le - \left( {\left( {1 - \frac{{S^{0} }}{{S_{n + 1} }}} \right)\left( {{\uppi } - \beta S_{n + 1} I_{n} - \upsilon S_{n + 1} + \varpi R_{n} } \right) + \left( {1 - \frac{{E_{n } }}{{E_{n + 1} }}} \right)\varphi_{1} \left( {\beta S_{n + 1} I_{n} - \left( {\delta + \sigma + \upsilon + \tau_{1} } \right)E_{n + 1} } \right)} \right. \\ & \quad + \left( {1 - \frac{{I_{n } }}{{I_{n + 1} }}} \right)\varphi_{2} \left( {\delta E_{n + 1} - \left( {\phi + \upsilon + \varepsilon } \right)I_{n + 1} } \right) + \left( {1 - \frac{{H_{n} }}{{H_{n + 1} }}} \right)\varphi_{3} \left( {\phi I_{n + 1} - \left( {t_{2} + \varepsilon + \upsilon } \right)H_{n + 1} } \right) \\ & \quad \left. { + \left( {1 - \frac{{R_{n} }}{{R_{n + 1} }}} \right)\varphi_{4} \left( {\tau_{1} E_{n + 1} + t_{2} H_{n + 1} - \left( {\varpi + \upsilon } \right)R_{n + 1} } \right)} \right). \\ \end{aligned}$$

**Figure 3:**
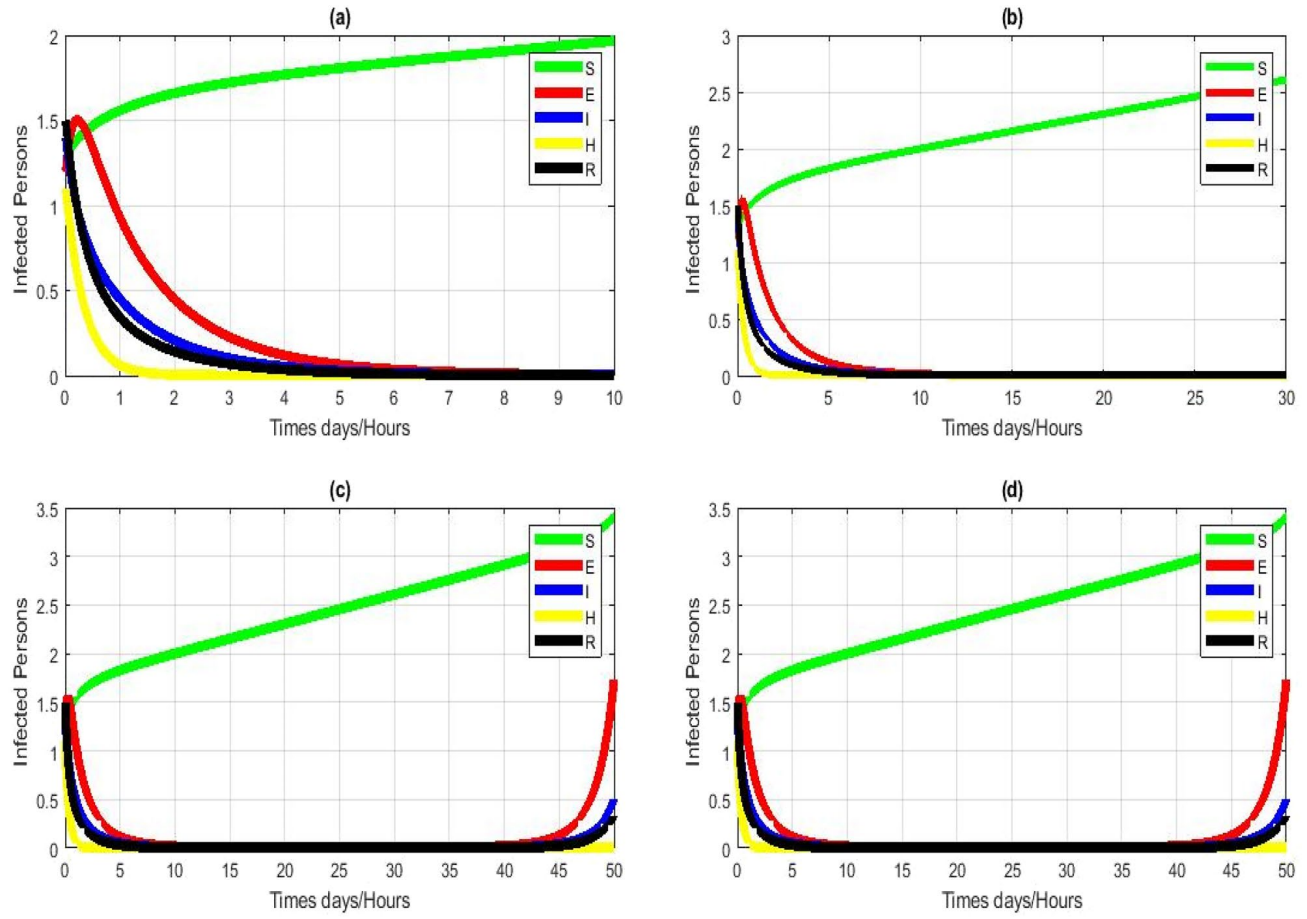
Numerical simulation for model (1) by using NSFD scheme with $\left(\mathbf{a}\right) h= 0.01,\left(\mathbf{b}\right) h = 0.1,\left(\mathbf{c}\right) h=1,\left(\mathbf{d}\right) h=10$. (**a**–**d**) Stable CEE point with $\uppi =5$ other parameters remain fixed $\beta =0.00000618$, ${\tau }_{1}=0.64505$, $\theta =0.02189 \upsilon =0.4995$, $\varpi =0.3232$, $\delta =0.002981,\mathcal{E}=0.38974$, ${t}_{2}=0.06813$.

Figure 3 a–d ${R}_{0}\ge 1$ this fig shows that the system is divergent at CEE point ${E}^{*}$ for NSFD scheme (4). The NSFD scheme is hence divergent for model (1) for all finite step sizes. Subfigures (a-d) show results for the concinnity of the primary susceptible $S\left(t\right)$, exposed $E\left(t\right)$, Infected persons $I\left(t\right)$, hospitalized persons $H\left(t\right)$, and recovered $R\left(t\right)$. The simulations were performed with the parameter values ​​shown in Table 1. The number of reproductions is ${R}_{0}\ge 1$.

Simple calculations yield

18 $$\begin{aligned} \Delta Q_{n} & \le - \left( {\left( {1 - \frac{{S^{0} }}{{S_{n + 1} }}} \right)\left( {{\uppi } + \phi_{1} \beta S_{n + 1} I_{n} - \left( {1 - \frac{{I_{n} }}{{I_{n + 1} }}} \right)\phi_{1} \left( {\delta + \sigma + \upsilon + \tau_{1} } \right)E_{n + 1} E_{n + 1} } \right) + \left( {1 - \frac{{E_{n } }}{{E_{n + 1} }}} \right)} \right. \\ & \quad \phi_{2} \beta S_{n + 1} I_{n} + \phi_{2} \delta E_{n + 1} + \phi_{3} \upsilon I_{n} + \phi_{3} \varepsilon H_{n} + \left( {1 - \frac{{H_{n } }}{{H_{n + 1} }}} \right)\phi_{3} \left( {\varepsilon + \upsilon } \right)H_{n + 1} + \phi_{4} \upsilon E_{n} + \phi_{4} \varphi H_{n} \\ & \quad \left. { + \left( {1 - \frac{{R_{n} }}{{R_{n + 1} }}} \right)\phi_{4} \left( {\varpi + \upsilon } \right)R_{n + 1} } \right). \\ & \le - \left( {1 - \frac{{S_{0 } }}{{S_{n + 1} }}} \right)\left( {{\uppi } - \left( {1 - \frac{{I_{n} }}{{I_{n + 1} }}} \right)\phi_{1} \left( {\delta + \sigma + \upsilon + \tau_{1} } \right)E_{n + 1} + \left( {1 - \frac{{E_{n } }}{{E_{n + 1} }}} \right) \phi_{2} \beta S_{n + 1} I_{n} } \right. \\ & \quad \left. {\left. { - \left( {1 - \frac{{H_{n } }}{{H_{n + 1} }}} \right)\phi_{3} \left( {\varepsilon + \upsilon } \right)H_{n + 1} + \left( {1 - \frac{{R_{n} }}{{R_{n + 1} }}} \right)\phi_{4} \varphi H_{n} } \right)} \right) \\ \end{aligned}$$

As ${S}^{0}=\frac{\uppi }{\upsilon }$ which implies ${S}^{0}\upsilon =\uppi$. By substituting $\uppi$ in (18), we obtain

$$\begin{aligned} \Delta X_{n} & \le - \left( {1 - \frac{{S^{0} }}{{S_{n + 1} }}} \right)\left( {S^{0} \upsilon - \upsilon S_{n + 1} - \left( {1 - \frac{{I_{n} }}{{I_{n + 1} }}} \right)\varphi_{1} \left( {\delta + \sigma + \upsilon + \tau_{1} } \right)E_{n + 1} } \right. \\ & \quad \left. { + \left( {1 - \frac{{E_{n } }}{{E_{n + 1} }}} \right) \varphi_{2} pS_{n} - \left( {1 - \frac{{H_{n } }}{{H_{n + 1} }}} \right)\varphi_{3} \left( {\varepsilon + \upsilon } \right)H_{n + 1} + \left( {1 - \frac{{R_{n} }}{{R_{n + 1} }}} \right)\varphi_{4} \varphi H_{n} } \right). \\ & = \frac{ - \upsilon }{{S_{n + 1} }}(\left( {S_{n + 1} - S^{0} } \right)^{2} - \left( {1 - \frac{{I_{n} }}{{I_{n + 1} }}} \right)\varphi_{1} \left( {\delta + \sigma + \upsilon + \tau_{1} } \right)E_{n} + \varphi_{2} \frac{{\beta \delta {\uppi }}}{{\upsilon \left( {\phi + \upsilon + \varepsilon } \right)\left( {\delta + \sigma + \upsilon + \tau_{1} } \right)}}R_{0} \\ & \quad - \varphi_{3} \left( {\varepsilon + \upsilon } \right)H_{n + 1} + \varphi_{4} H_{n} ). \\ \end{aligned}$$

Let $${C}_{1}=\frac{\beta \delta\uppi }{\upsilon \left(\phi +\upsilon +\varepsilon \right)\left(\delta +\sigma +\upsilon +{\tau }_{1}\right)}$$

19 $$=\frac{-\upsilon }{{S}_{n+1}}{(\left({S}_{n+1}-{S}^{0}\right)}^{2}-\left(1-\frac{{I}_{n}}{{I}_{n+1}}\right){\varphi }_{1}\left(\delta +\sigma +\upsilon +{\tau }_{1}\right){E}_{n}+{ \varphi }_{2}{C}_{1}{R}_{0}-{ \varphi }_{3}(\varepsilon +\upsilon ){H}_{n+1}+{\varphi }_{4}{H}_{n}).$$

Hence, if ${R}_{0}\le 1$ then from (19) employs $\Delta {X}_{n}\le 0$ for all $n\ge 0$. Therefore, ${X}_{n}$ is a non-increasing sequence. So, here arises as a constant ${\text{O}}$ such that ${{\text{lim}}}_{n\to \infty }{X}_{n}=X$ which recommends ${{\text{lim}}}_{n\to \infty }\left({X}_{n+1}-{X}_{n}\right)=0$. From system (3) and ${{\text{lim}}}_{n\to \infty }\Delta {X}_{n}=0$ we have ${{\text{lim}}}_{n\to \infty }{S}_{n}={S}^{0}$. For the case ${R}_{0}<1,$ we have ${{\text{lim}}}_{n\to \infty }{S}_{n+1}={S}^{0}$ and ${{\text{lim}}}_{n\to \infty }{E}_{n}=0,{{\text{lim}}}_{n\to \infty }{I}_{n}=0.$ From system (3), we attain ${{\text{lim}}}_{n\to \infty }{E}_{n}=0,{{\text{lim}}}_{n\to \infty }{R}_{n}=0$ and ${{\text{lim}}}_{n\to \infty }{H}_{n}=0.$ For the case ${R}_{0}=1,$ we have ${{\text{lim}}}_{n\to \infty }{S}_{n+1}={S}^{0}.$ Thus, from system (3), we obtain ${{\text{lim}}}_{n\to \infty }{R}_{n}=0,{{\text{lim}}}_{n\to \infty }{H}_{n}=0,{{\text{lim}}}_{n\to \infty }{E}_{n}=0$ , ${{\text{lim}}}_{n\to \infty }{I}_{n}=0$ and ${{\text{lim}}}_{n\to \infty }{I}_{n}=0$. Hence, ${E}_{0}$ is GAS.

#### Theorem 3

For all $\psi >0$, the CEE is GAS for NSFD model (4) when ${R}_{0}>1$.

#### Proof

Let us define.

$${W}_{n}\left({S}_{n}{, E}_{n},{I}_{n},{H}_{n},{R}_{n}\right)={S}^{*}M\left(\frac{{S}_{n}}{{S}^{*}}\right)+{\phi }_{1}{E}^{*}M\left(\frac{{E}_{n}}{{E}^{*}}\right)+{\phi }_{2}{Q}^{*}M\left(\frac{{I}_{n}}{{I}^{*}}\right){+\phi }_{3}{H}^{*}M\left(\frac{{H}_{n}}{{H}^{*}}\right){+\phi }_{4}{R}^{*}M\left(\frac{{R}_{n}}{{R}^{*}}\right),$$

where ${\phi }_{i}>0,i=\mathrm{1,2},\mathrm{3,4}$ which we will use later. It is clear that ${W}_{n}\left({S}_{n}{, E}_{n},{I}_{n},{H}_{n},{R}_{n}\right)>0$ for all ${S}_{n}>0, {E}_{n}>0,{I}_{n}>0, {H}_{n}>0, {R}_{n}>0$ and ${W}_{n}\left({S}^{*},{E}^{*},{I}^{*},{H}^{*}, {R}^{*}\right)=0.$

Where ${\phi }_{i}$ is a parameter that can be varied depending on the specific needs of the system, and different values of ${\phi }_{i}$ are chosen according to these needs. ${W}_{n}\left({S}^{*},{E}^{*},{I}^{*},{H}^{*}, {R}^{*}\right)>0$ because we have shown that this sequence is monotonically increasing. When ${W}_{n}\left({S}^{*},{E}^{*},{I}^{*},{H}^{*}, {R}^{*}\right)>1$, the system diverges, indicating widespread disease spread. Conversely, when ${W}_{n}\left({S}^{*},{E}^{*},{I}^{*},{H}^{*}, {R}^{*}\right)=0$, the system reaches a saddle point, resulting in no disease spread. Therefore, for realistic and meaningful representations of the system, we choose $0<{W}_{n}\left({S}^{*},{E}^{*},{I}^{*},{H}^{*}, {R}^{*}\right)\le 1$.

Let us take

$$\Delta {W}_{n}={W}_{n+1}-{w}_{n},$$

we get

20 $$\begin{aligned} \Delta W_{n} & = S^{*} M\left( {\frac{{S_{n + 1} }}{{S^{*} }}} \right) + \phi_{1} E^{*} M\left( {\frac{{E_{n + 1} }}{{E^{*} }}} \right) + \phi_{2} I^{*} M\left( {\frac{{I_{n + 1} }}{{I^{*} }}} \right) + \phi_{3} H^{*} M\left( {\frac{{H_{n + 1} }}{{H^{*} }}} \right) + \phi_{4} R^{*} M\left( {\frac{{R_{n + 1} }}{{R^{*} }}} \right) + \\ & \quad - \left[ {S^{*} M\left( {\frac{{S_{n} }}{{S^{*} }}} \right) + \phi_{1} E^{*} M\left( {\frac{{E_{n} }}{{E^{*} }}} \right) + \phi_{2} I^{*} M\left( {\frac{{I_{n} }}{{I^{*} }}} \right) + \phi_{3} H^{*} M\left( {\frac{{H_{n} }}{{H^{*} }}} \right) + \phi_{4} R^{*} M\left( {\frac{{R_{n} }}{{R^{*} }}} \right)} \right]. \\ & = S^{*} \left( {\frac{{S_{n + 1} }}{{S^{*} }} - \frac{{S_{n} }}{{S^{*} }} + {\text{ln}}\frac{{S_{n } }}{{S_{n + 1} }}} \right) + \phi_{1} E^{*} \left( {\frac{{E_{n + 1} }}{{E^{*} }} - \frac{{E_{n} }}{{E^{*} }} + {\text{ln}}\frac{{E_{n} }}{{E_{n + 1} }}} \right) + \phi_{2} H^{*} \left( {\frac{{H_{n + 1} }}{{H^{*} }} - \frac{{H_{n} }}{{H^{*} }} + {\text{ln}}\frac{{H_{n} }}{{H_{n + 1} }}} \right) \\ & \quad + \phi_{3} I^{*} \left( {\frac{{I_{n + 1} }}{{I^{*} }} - \frac{{I_{n} }}{{I^{*} }} + {\text{ln}}\frac{{I_{n} }}{{I_{n + 1} }}} \right) + \phi_{4} R^{*} \left( {\frac{{R_{n + 1} }}{{R^{*} }} - \frac{{R_{n} }}{{R^{*} }} + {\text{ln}}\frac{{R_{n} }}{{R_{n + 1} }}} \right). \\ \end{aligned}$$

By using inequality ${\text{ln}}Z\le Z-1$, (20) can be written as

21 $$\begin{aligned} \Delta W_{n} & \le S^{*} \left( {\frac{{S_{n + 1} - S_{n} }}{{S^{*} }} + \frac{{S_{n } }}{{S_{n + 1} }} - 1} \right) + \phi_{1} E^{*} \left( {\frac{{E_{n + 1} - E_{n} }}{{E^{*} }} + \frac{{E_{n} }}{{E_{n + 1} }} - 1} \right) + \phi_{2} I^{*} \left( {\frac{{I_{n + 1} - I_{n} }}{{I^{*} }} + \frac{{I_{n} }}{{I_{n + 1} }} - 1} \right) \\ & \quad + \phi_{3} H^{*} \left( {\frac{{H_{n + 1} - H_{n} }}{{H^{*} }} + \frac{{H_{n} }}{{H_{n + 1} }} - 1} \right) + \phi_{4} R^{*} \left( {\frac{{R_{n + 1} - R_{n} }}{{R^{*} }} + \frac{{R_{n} }}{{R_{n + 1} }} - 1} \right). \\ & = \left( {1 - \frac{{S^{*} }}{{S_{n + 1} }}} \right)\left( {S_{n + 1} - S_{n} } \right) + \phi_{1} \left( {1 - \frac{{E^{*} }}{{E_{n + 1} }}} \right)\left( {E_{n + 1} - E_{n} } \right) + \phi_{2} \left( {1 - \frac{{I^{*} }}{{I_{n + 1} }}} \right)\left( {I_{n + 1} - I_{n} } \right) \\ & \quad + \phi_{3} \left( {1 - \frac{{H^{*} }}{{H_{n + 1} }}} \right)\left( {H_{n + 1} - H_{n} } \right) + \phi_{4} \left( {1 - \frac{{R^{*} }}{{R_{n + 1} }}} \right)\left( {R_{n + 1} - R_{n} } \right) \\ \end{aligned}$$

By applying system (3), (21) becomes

22 $$\begin{aligned} \Delta W_{n} & \le \left( {1 - \frac{{S^{*} }}{{S_{n + 1} }}} \right)\left( {{\uppi } - \beta S_{n + 1} I_{n} - \upsilon S_{n + 1} + \varpi R_{n} } \right) + \phi_{1} \left( {1 - \frac{{E^{*} }}{{E_{n + 1} }}} \right)\beta S_{n + 1} I_{n} - \left( {\delta + \sigma + \upsilon + \tau_{1} } \right)E_{n + 1} \\ & \quad + \phi_{3} \left( {1 - \frac{{I^{*} }}{{I_{n + 1} }}} \right)\left( {\delta E_{n + 1} - \left( {\phi + \upsilon + \varepsilon } \right)I_{n + 1} } \right) + \phi_{4} \left( {1 - \frac{{H^{*} }}{{H_{n + 1} }}} \right)\left( {\phi I_{n + 1} - \left( {t_{2} + \varepsilon + \upsilon } \right)H_{n + 1} } \right) \\ & \quad + \phi_{2} \left( {1 - \frac{{R^{*} }}{{R_{n + 1} }}} \right)\left( {\tau_{1} E_{n + 1} + t_{2} H_{n + 1} - \left( {\varpi + \upsilon } \right)R_{n + 1} } \right) \\ \end{aligned}$$

By replacing $\uppi =\beta {S}^{*}{I}^{*}+\upsilon {S}^{*}-\varpi {R}^{*}$ in (22), we obtain

23 $$\begin{aligned} \Delta W_{n} & \le \left( {1 - \frac{{S^{*} }}{{S_{n + 1} }}} \right)\left( {\beta S^{*} I^{*} + \upsilon S^{*} - \varpi R^{*} - \beta S_{n + 1} I_{n} - \upsilon S_{n + 1} + \varpi R_{n} } \right) + \phi_{1} \left( {1 - \frac{{E^{*} }}{{E_{n + 1} }}} \right)\left( {\beta SI - \left( {\delta + \sigma + \upsilon + \tau_{1} } \right)E^{*} } \right) \\ & \quad + \phi_{3} \left( {1 - \frac{{I^{*} }}{{I_{n + 1} }}} \right)\left( {\delta E^{*} - \left( {\phi + \upsilon + \varepsilon } \right)I^{*} } \right) + \phi_{4} \left( {1 - \frac{{H^{*} }}{{H_{n + 1} }}} \right)\left( {\phi I^{*} - \left( {t_{2} + \varepsilon + \upsilon } \right)H^{*} } \right) \\ & \quad + \phi_{2} \left( {1 - \frac{{R^{*} }}{{R_{n + 1} }}} \right)\left( {\tau_{1} E^{*} + t_{2} H^{*} - \left( {\varpi + \upsilon } \right)R^{*} } \right). \\ & = \left( {1 - \frac{{S^{*} }}{{S_{n + 1} }}} \right)\left( {\upsilon S^{*} - \upsilon S_{n + 1} } \right) + \phi_{1} \left( {1 - \frac{{E^{*} }}{{E_{n + 1} }}} \right)\left( {\beta SI - \left( {\delta + \sigma + \upsilon + \tau_{1} } \right)E^{*} ) } \right) + \phi_{2} \left( {1 - \frac{{I^{*} }}{{I_{n + 1} }}} \right)\left( {\delta E^{*} - \left( {\phi + \upsilon + \varepsilon } \right)I^{*} } \right) \\ & \quad + \phi_{3} \left( {1 - \frac{{H^{*} }}{{H_{n + 1} }}} \right)\left( {\phi I^{*} - \left( {t_{2} + \varepsilon + \upsilon } \right)H^{*} } \right) + \phi_{4} \left( {1 - \frac{{R^{*} }}{{R_{n + 1} }}} \right)\left( {\tau_{1} E^{*} + t_{2} H^{*} - \left( {\varpi + \upsilon } \right)R^{*} } \right) \\ \end{aligned}$$

By substituting $\beta {S}^{*}{ I}^{*}={\phi }_{1}\left(\delta +\sigma +\upsilon +{\tau }_{1}\right){E}^{*}$,$\delta {E}^{*}={\phi }_{2}\left(\phi +\upsilon +\varepsilon \right){I}^{*}$,$\phi {I}^{*}={\phi }_{3}\left({t}_{2}+\varepsilon +\upsilon \right){H}^{*}$,${\tau }_{1}{E}^{*}+{t}_{2}{H}^{*}={\phi }_{4}\left(\varpi +\upsilon \right){R}^{*}$ in (23), we get

$$\begin{aligned} \Delta V_{n} & \le \frac{ - \upsilon }{{S_{n + 1} }}\left( {S_{n + 1} - S^{*} } \right)^{2} + \left( {1 - \frac{{S^{*} }}{{S_{n + 1} }}} \right)\left( {\delta + \sigma + \upsilon + \tau_{1} } \right)E^{*} - \left( {\theta + \delta + \upsilon + \varrho } \right)E^{*} \frac{{S_{n + 1} R_{n} E^{*} }}{{S^{*} E_{n + 1} }} + \\ & \quad - \phi_{2} \delta E^{*} \frac{{S_{n + 1} R_{n} A^{*} }}{{S^{*} R_{n}^{*} I_{n + 1} }} - \phi_{1} vS^{*} + \beta S^{*} I^{*} \frac{{I^{*} E_{n + 1} }}{{I_{n + 1} E^{*} }} + \phi_{2} \left( {\varepsilon + \upsilon } \right)I^{*} - \phi_{3} \phi I^{*} \frac{{S_{n + 1} R_{n} H^{*} }}{{S^{*} H_{n + 1} }} \\ & \quad + \phi_{3} \phi I^{*} - \phi_{2} \left( {\varepsilon + \upsilon } \right)I^{*} \frac{{R^{*} R_{n + 1} }}{{I_{n + 1} I_{n}^{*} }} - \phi_{3} \phi H^{*} \frac{{R^{*} I^{*} }}{{R_{n + 1} }}. \\ & = \frac{ - \upsilon }{{S_{n + 1} }}\left( {S_{n + 1} - S^{*} } \right)^{2} - \phi_{1} H^{*} \left( {M\left( {\frac{{S^{*} }}{{S_{n + 1} }}} \right) + M\left( {\frac{{S_{n + 1} R_{n} E^{*} }}{{S^{*} R^{*} E_{n + 1} }}} \right) + M\left( {\frac{{I^{*} E_{n + 1} }}{{I_{n + 1} E^{*} }}} \right) + H\left( {\frac{{R^{*} I_{n + 1} }}{{R_{n + 1} I^{*} }}} \right)} \right) \\ & \quad - \phi_{2} \delta E^{*} \left( {M\left( {\frac{{S^{*} }}{{S_{n + 1} }}} \right) + M\left( {\frac{{S_{n + 1} R_{n} R^{*} }}{{S^{*} I^{*} R_{n + 1} }}} \right) + H\left( {\frac{{R^{*} R_{n + 1} }}{{H_{n + 1} I^{*} }}} \right)} \right) - \phi_{3} \phi H^{*} \left( {M\left( {\frac{{S^{*} }}{{S_{n + 1} }}} \right) + M\left( {\frac{{S_{n + 1} R_{n} H^{*} }}{{S^{*} R^{*} H_{n + 1} }}} \right) + M\left( {\frac{{H_{n + 1} R^{*} }}{{R^{*} R_{n + 1} }}} \right)} \right). \\ \end{aligned}$$

Thus, $W_{n}$ is an increasing sequence and $\exists W\parallel {\text{lim}}_{n \to \infty } W_{n} = W$. Therefore,${\text{lim}}_{n \to \infty } \Delta W_{n} = 0 \Rightarrow {\text{lim}}_{n \to \infty } S_{n} = S^{*}$, ${\text{lim}}_{n \to \infty } E_{n} = E^{*}$, ${\text{lim}}_{n \to \infty } I_{n} = Q^{*}$, ${\text{lim}}_{n \to \infty } H_{n} = H^{*} ,{\text{ lim}}_{n \to \infty } R_{n} = R^{*}$.

## Conclusions

This study utilized a novel continuous mathematical model incorporating both symptomatic and asymptomatic individuals to unveil the key drivers of COVID-19 transmission dynamics. We identified a critical threshold value ${R}_{0}$, the basic reproduction number governing the stability of disease-free and endemic states. Our innovative Numerical Solution by Non-Standard Finite Differences (NSFD) scheme ensured accurate and efficient results with finite step sizes while maintaining both mathematical and biological plausibility. Additionally, we established the boundedness and positivity of solutions within the NSFD framework. By applying diverse stability criteria under the NSFD paradigm, we comprehensively analyzed various disease states.

Moving forward, we will compare the dynamic reliability of our discrete model against its continuous counterpart across various time steps. Furthermore, we will conduct numerical simulations to showcase the qualitative accuracy and efficiency of the NSFD approach. Beyond validation, we plan to explore the adaptability of NSFD to other generalized epidemic models, leading to broader insights into disease propagation dynamics. To delve deeper into system dynamics, we aim to incorporate sensitivity analysis alongside NSFD to identify key parameters influencing disease spread. These advanced methodologies promise to refine our models and illuminate the intricate mechanisms governing outbreaks.

## Data Availability

The data used to support the finding of this study are included within the article.
